# A novel directed evolution platform for engineering chemically gated protein switches

**DOI:** 10.1039/d5cc06861d

**Published:** 2026-02-24

**Authors:** Luis A. Vázquez-Rivera, Jiaqi Shen, Gwendolyn Shingles, Guanwei Zhou, Wenjing Wang

**Affiliations:** a Life Sciences Institute, University of Michigan Ann Arbor Michigan USA wenjwang@umich.edu; b Program in Chemical Biology, University of Michigan Ann Arbor Michigan USA; c Department of Chemistry, University of Michigan Ann Arbor Michigan USA

## Abstract

Chemically gated protein switches have broad applications in regulating cellular activities. In this study, we introduce DuoSelect, a yeast surface selection platform for selecting protein switches based on signal-to-background ratios by combining both negative and positive selections in a single round of sorting. We demonstrate this platform's utility by selecting for two chemical-dependent protein switches and showcase the protein switch's application in cellular assays.

## Abstract 2

DuoSelect is a yeast surface display platform for rapid isolation of chemically gated protein switches. It integrates positive and negative selection in one round by two-color fluorescence readout, enriching high signal-to-background variants.

## Graphical abstract

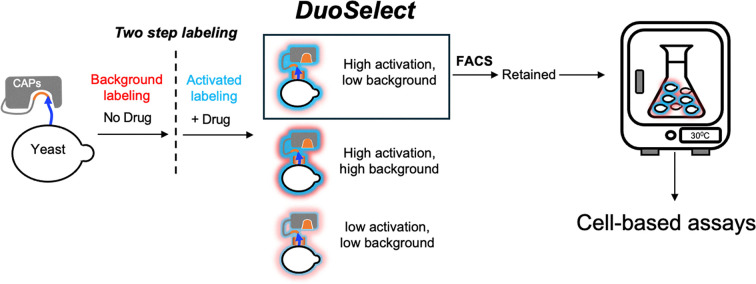

Optogenetic and chemogenetic tools have been widely applied in biological studies to regulate cellular processes with defined temporal control. Their designs heavily rely on efficient protein switches that can be toggled between inactive and activated states by light^1–5^ or small molecules.^6–9^ The design of new protein switches remains a challenging process.

To achieve high dynamic range for protein switches, directed evolution has been applied to reduce background in the inactive state and increase the signal in the activated state. A sequential negative selection followed by positive selection, without library amplification, could be performed using phage display. This strategy has been applied to design various chemogenetic and optogenetic switches (*e.g.*, AbCID,^10^ nanoCLAMP,^11^ COMBINES-CID,^12^ and iLID^13^). Conversely, yeast display has also been used to optimize protein switches. Alternating positive and negative selections were applied, but yeast library amplification occurs between each selection. Yeast display strategy has enabled the development of the photo-switchable enzyme LOV-Turbo,^14^ photo-switchable enzyme substrate eLOV-TEVcs,^15^ and chemical-switchable peptides CapN-TEVcs and SsrA-CapC.^7^

Because sequential negative and positive selections only allow selection based on one parameter (background or activation signal) at a time, the process is prone to retaining suboptimal clones. For instance, negative selections may select false negatives: clones that have low background but also low activation signals. These must be removed in a subsequent positive selection round. Similarly, positive selection can select false positives - clones with high activation signals but also high background. Therefore, multiple rounds of alternating selection are necessary to effectively enrich for clones with a high signal-to-background ratio (SBR).

As an alternative to single-parameter selection methods, we present DuoSelect, a new yeast cell surface selection platform. DuoSelect represents the first protein-switch selection platform designed to directly select clones based on their SBRs for each yeast cell. To illustrate its utility for protein switches, we applied DuoSelect to two chemically activated protein domains.

Yeast surface display is highly versatile and enables multi-color labeling, allowing selection based on multiple parameters. To enable protein-switch selection based on both the activation and background signals on a single yeast cell, we harnessed the strength of yeast surface display to develop DuoSelect. To demonstrate the platform's application, we set out to select new versions of our previously-designed chemical-dependent protein switches, CapC and CapN (CAPs), that cage the C- and N-terminal portions of a peptide, respectively.^7^

As shown in Fig. 1, we used CapC to cage SsrA (an 8-amino acid peptide tag). To measure CapC activity, we designed an assay using SsrA's binding partner, SspB, fused to the enhanced Ascorbate Peroxidase 2 (APEX2). APEX2 performs proximity-dependent biotinylation, converting the transient SsrA-SspB binding into a covalent biotin label. The SspB-APEX2 labeling was performed first without shield-1 for background and then with shield-1 for activated signal labeling. After APEX2 labeling, the covalently attached biotin was labeled with streptavidin (strep) fused to a fluorophore—phycoerythrin (PE, background) and Alexa Fluor 647 (abbreviated as 647, activated signal). To ensure that the covalently labeled biotin from the background step is fully bound to Strep-PE and marked with Strep-647 in the activated labeling step, an excess amount of Strep-PE was used. Additionally, since streptavidin has a very high binding affinity for biotin (in the range of 10^−14^–10^−15^ M), its binding to biotin is considered irreversible, and therefore, the background strep-PE signal should be stable once bound and will not be replaced by strep-647 used in the activated labeling step.

**Fig. 1:**
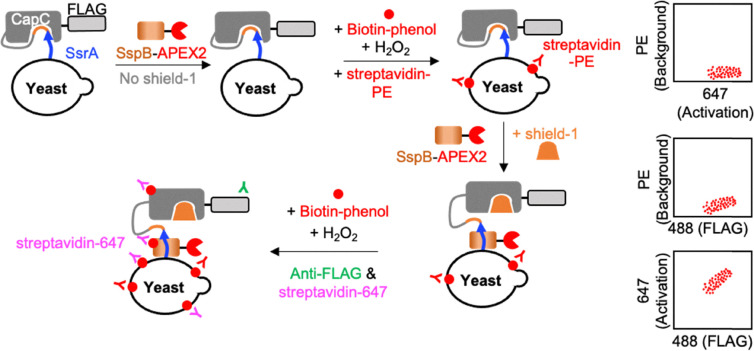
DuoSelect platform schematics. In DuoSelect, the binding assay with “no shield-1” and “+ shield-1” conditions are sequentially performed on the same yeast cell. In the leakage (background activity) labeling step, SspB-APEX2 fusion protein was incubated with yeast with “no shield-1”, followed by APEX-dependent proximity labeling. Covalently attached biotin molecules on the yeast surface are labeled with excess streptavidin-PE to ensure saturation. The resulting yeasts then undergo activation labeling, first by treatment with the SspB-APEX2 fusion protein and shield-1, followed by the APEX reaction. The newly attached biotin molecules are then labeled with streptavidin-Alexa Fluor 647 (647). This allows the two signals to be distinguished: the PE signal represents the leakage (background), while the 647 signal represents activation.

To verify the stability of strep-PE binding, we sequentially labeled cells with Strep-PE followed by strep-647 (Fig. S1). Consistent PE signals and negligible 647 binding (∼3%, matching background controls) confirmed that displacement did not occur. Therefore, we selected clones exhibiting high activation (647) and low background (PE) (Fig. S1, top FACS plot).

To evaluate the potential impact of hydrogen peroxide on fluorescence signals, we conducted a control experiment in which cells labeled with Strep-PE were exposed to hydrogen peroxide for 2 minutes (Fig. 2A). We compared H_2_O_2_ treatments (alone, with biotin, or with APEX2 incubation) against a PBSB control. Fluorescence remained consistent across all samples, confirming that short exposure does not diminish the Strep-PE signal (Fig. 2B). We first tested DuoSelect's ability to separate yeast cells with high and low SBRs using mixed populations of known positive and negative variants. For positive cells, we used our previously designed CapN1.0^7^ which controls SsrA activity in a shield-1-dependent manner. For negative cells, we used the pre-evolution CapN0^7^ which exhibits no caging effect on SsrA. Yeast cells expressing CapN1.0 and CapN0 were mixed in different ratios and labeled using the platform as shown in Fig. 1. When the positive and negative cells were mixed at 1 : 10 ratio, the two populations were clearly separated (Fig. S3), indicating that the DuoSelect method can separate constructs with different SBRs. Further, the ratio of the two separate populations falling into the positive and negative gates were calculated at 1 : 7, close to the mixing ratio of 1 : 10. However, when the cells were mixed at a ratio of 1 : 1000 and 1 : 10 000, the cells falling into the positive and negative cell gates in FACS analysis were at a ratio of 1 : 100 and 1 : 50, respectively, much higher than the mixing ratios. The higher frequency of the cells falling into the positive cell gate (high 647/PE ratios) are due to false positive cells that exhibit abnormally high strep-647 signal (Fig. S4), rather than due to the reduced background strep-PE signal. The abnormally high strep-647 signals are well above the signals of the main population of cells in the activated state (Fig. S4). We hypothesize that these signals arise from non-specific binding of strep-647 (Fig. S1) and from increased stickiness of dead or compromised cells during to the two-step labeling procedure.

**Fig. 2:**
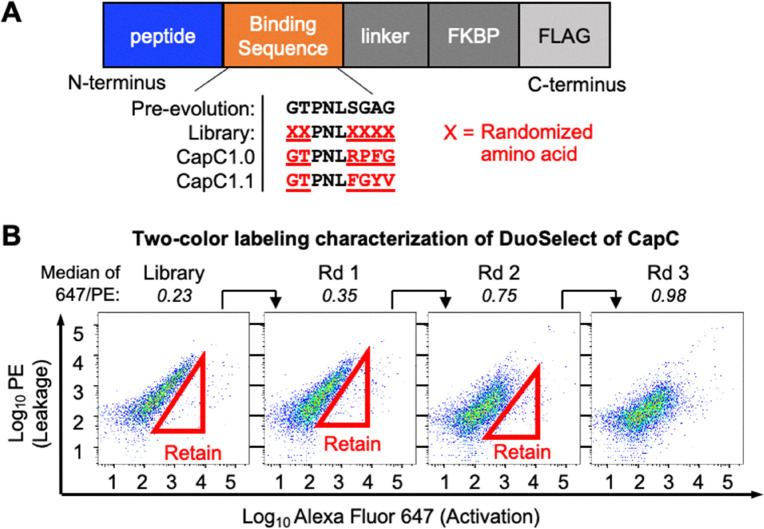
CapC evolution with DuoSelect. (A) The binding peptide sequences of CapC variants. (B) FACS analysis characterization of the CapC library selection using DuoSelect.

To avoid the above false positives in DuoSelect, we implemented a consecutive triple-gating strategy as shown in Fig. S4. Gate 1 selects the cells with low background signals and high expression levels. Gate 2 selects the cells that fall into the main population of cells in the activation signal plot, excluding the false positives exhibiting abnormally high 647 signals (Fig. S4). Gate 3 selects for cells with high 647/PE ratios (SBRs).

To demonstrate the efficiency of DuoSelect in selecting protein switches, we performed a side-by-side comparison of DuoSelect (Fig. 1) with the conventional single-color alternating negative and positive selections, referred to as SingleSelect in this paper (Fig. S5). We used an existing CapC library that has six positions randomized (Fig. 2A), and was previously used to evolve CapC1.0. For DuoSelect, three rounds of selections were performed according to the scheme shown in Fig. 2B to enrich the cells with high 647/PE ratios, approximating high SBRs. The 647/PE signal ratios increased after each round of selection (Fig. 2B). In SingleSelect, we performed three rounds of alternating negative and positive selections (Fig. 3A, left).

**Fig. 3:**
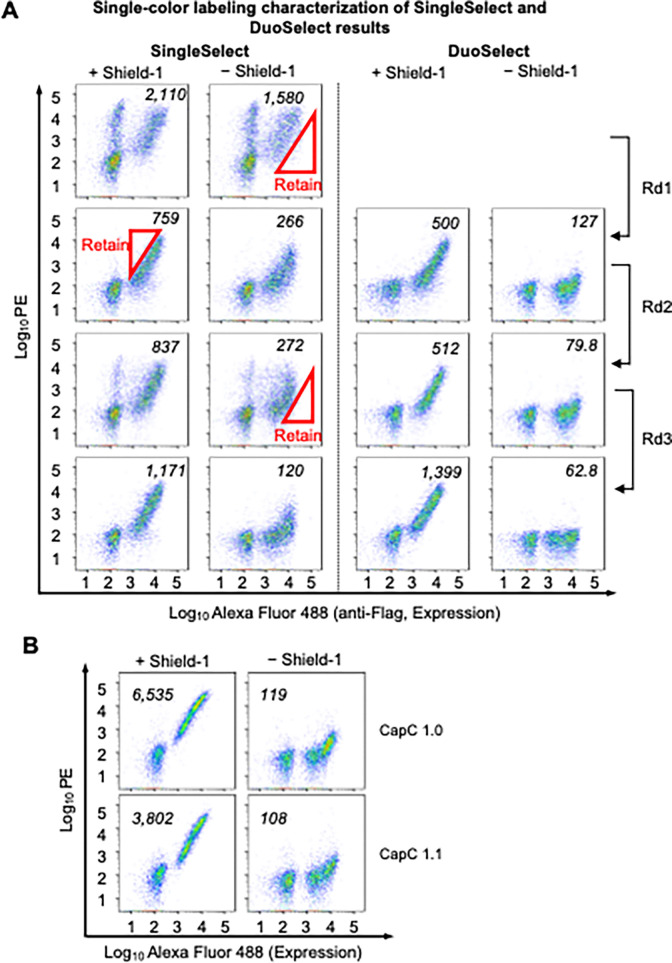
FACS analysis of DuoSelect and SingleSelect. (A) Single-color labeling characterization of CapC library selection results using SingeSelect and DuoSelect, and (B) of CapC 1.0 and the evolved CapC 1.1 from DuoSelect. Numbers indicate median of expression-positive population.

We then compared DuoSelect and SingleSelect rounds using single-color labeling characterization (Fig. 3A). After the first round, yeast cells enriched by DuoSelect already exhibited a lower median background signal (PE signal under no shield-1 condition) than those enriched by SingleSelect. In subsequent rounds, DuoSelect led to a desirable gradual reduction in background signal (Fig. 3A), while SingleSelect showed an increase in median background PE signal after positive selection. This increase in SingleSelect reflects the enrichment of false positives, necessitating an additional negative selection round to reduce background. Overall, this study highlights DuoSelect as a more efficient approach for selecting clones with high SBRs, enabling the identification of desired mutants in fewer steps than conventional methods.

DuoSelect efficiently isolated multiple chemically dependent variants from a predominantly non-responsive library (Fig. S6). Among these, CapC1.1 exhibited caging activity comparable to the previously selected CapC1.0; this performance similarity is expected, as both variants originated from the same starting library (Fig. 3B).

To evaluate CapC1.1 in a mammalian context, we characterized it alongside CapC1.0 using a mammalian two-hybrid (MTH) system (Fig. 4A), where Shield-1 induces SsrA–SspB interaction and subsequent firefly luciferase transcription. Consistent with yeast characterizations, CapC1.1 exhibited a signal-to-background ratio (SBR) of 12.8, a slight improvement over the 10.6 observed for CapC1.0 (Fig. 4B).

**Fig. 4:**
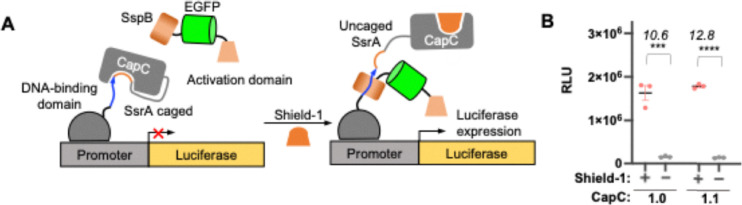
Characterization of evolved CapC variants in HEK293T. (A) Scheme of shield-1 induced gene transcription of luciferase. DNA-binding domain (DBD) is Gal4-DBD. Transcription-activation domain is VP16. (B) Luminescence analysis of the CapC-based transcriptional system depicted in a with CapC 1.0–1.5 under +/− shield-1 conditions. shield-1, 10 µM. The values on the plot are the luminescence signal ratio of the “+ shield-1” *versus* the “no shield-1” condition. *P* values are determined by unpaired two-tailed *t*-tests. **P < 0.01; ***P < 0.001; ****P < 0.0001; ns, not significant. Errors, s.e.m.

We next characterized CapC1.1's efficiency in caging a neuropeptide agonist for opioid receptors (ORs), [Met^5^]-enkephalin (hereafter referred to as enkephalin). We previously showed that CapC-caged enkephalin tethered to the N-terminal extracellular side of mu-OR (MOR) (Fig. S7A) could regulate MOR's activity in a shield-1 dependent manner. Similarly, to direct membrane trafficking without perturbing the function of enkephalin, a cleavable signal sequence (KTIIALSYIFCLVFA)^16^ was fused to the N-terminus of enkephalin.^7^ Enkephalin is expected to be caged under no shield-1 condition and therefore less active than in the presence of shield-1. The activity of MOR was characterized using GloSensor^17,18^ which indicates the level of the second messenger cAMP.

As shown in Fig. S7B, the addition of the agonist loperamide reduced cAMP level, suggesting that MOR is functional when fused with the CapC-enkephalin. The addition of shield-1 also induced a decrease in cAMP level for both CapC1.0 and CapC1.1 constructs, indicating that enkephalin was controlled by CapC in a shield-1-dependent manner. However, like our previous findings, the addition of naloxone increased the cAMP level for both constructs, highlighting that CapC1.0- and CapC1.1-caged enkephalin exhibit leaky activity.

To account for variations in biosensor expression, luminescence signals were normalized to the mean naloxone response at the final time point (Fig. S7C). Following normalization, both constructs displayed overlapping maximal activities; however, CapC1.1 exhibited 1.7-fold higher Shield-1 dependence and 15% lower leaky activity than CapC1.0. These results corroborate findings from yeast surface and MTH characterizations, confirming that CapC1.1 provides superior regulation of peptide activity.

We applied DuoSelect to evolve the CapN platform, generating a library of variants from CapN1.0 *via* targeted random mutagenesis of the four residues preceding the PNL (Fig. 5A). Three rounds of FACS sorting utilizing the consecutive triple gating strategy yielded a progressive increase in signal-to-background ratio (Fig. S3 and Fig. 5B); notably, the median 647/PE ratio rose from 0.3 in the initial pool to 35.9 after the third round. We then sequenced the enriched CapN clones and identified three CapN variants, named as CapN2.1-2.3 as indicated in Fig. 5C.

**Fig. 5:**
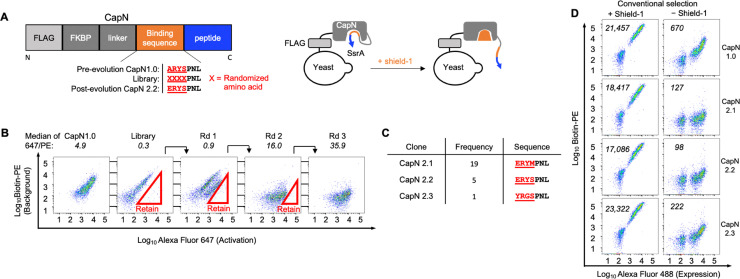
CapN selection using DuoSelect. (A) Schematics of the CapN DNA construct and CapN displayed on yeast surface. The binding sequences are shown for the pre-evolved (CapN1.0), the library and the best evolved clone (CapN2.2). (B) FACS selection of the CapN library in a using DuoSelect. Numbers above the FACS plot indicate the medium of the 647/PE ratios. (C) Frequency and sequence of three CapN clones enriched after DuoSelect evolution. (D) Single color labeling characterization of evolved CapN variants. The numbers within the FACS plot indicates the medium PE value of the population with positive FLAG signal.

Interestingly, despite the random mutagenesis of four amino acids in the CapN1.0 binding sequence to create the CapN library, the selected clones CapN2.1 and CapN2.2 showed variations in only two and one residue, respectively, when compared to CapN1.0. This conservation suggests that the RY residues within the binding sequences are functionally important for the interaction with the FKBP binding pocket.

We characterized CapN2.1-2.3 along with CapN1.0 on the yeast cell surface using the single-color labeling characterization for the conditions with and without shield-1 (Fig. 5C and D). CapN2.2 shows the lowest background signal in the labeling condition without shield-1, significantly lower than that of the original CapN1.0.

We next characterized the evolved CapN clones using the MTH system in HEK293T cells, like the application of CapC in Fig. 4A, except that CapN was used to cage the SsrA in place of CapC (Fig. S8A). Surprisingly, there was no significant improvement for CapN2.2 over the original CapN1.0 when evaluated using the MTH assay (Fig. S8B and C). CapN's differential performance likely stems from distinct host factors. The yeast surface display creates a higher effective molarity than HEK293T cytosolic expression. Additionally, differences in folding machinery between fungal and mammalian cells may alter the active protein fraction, affecting reporter output. While CapN2.1 did not show improved performance using the MTH system, its improved caging efficiency on yeast cell surface could still be advantageous for some applications. Therefore, we demonstrate that CapN2.2 exhibits a markedly improved SBR on yeast surface, even though this enhancement is not observed in mammalian HEK293T cells (Fig. S8).

In conclusion, to facilitate the selection of protein switches, we developed DuoSelect, a novel yeast display platform that characterizes both background and activation signals within a single selection round. Compared to SingleSelect, DuoSelect demonstrated superior efficiency in designing chemically dependent CapC variants. This strategy is broadly applicable for improving various protein switches exhibiting state-dependent signals. DuoSelect enabled the isolation of CapC1.1 from a non-responsive library and the engineering of the improved CapN2.2. In summary, DuoSelect represents the first selection platform capable of directly screening protein switches based on their SBRs, offering a powerful tool for future switch design.

## Author contributions

L. V., J. S., G. S. and W. W. designed and analyzed CAPs experiments. L. V. and J. S. performed the CAPs evolution and characterization. G. S. performed CapC characterization using MTH system. G. Z. performed CapC characterization in opioid peptide and some of the CapN FACS sorting. L. V., J. S., G. S., and W. W. designed cell culture experiments. L. V., performed all other cell culture application and characterization. All authors analyzed data, wrote, and edited the manuscript.

## Conflicts of interest

The authors declare no conflict of interests.

## Supplementary Material

CC-062-D5CC06861D-s001

## Data Availability

All the DNA constructs used in this study are available upon request to the corresponding author. All the data supporting the findings of this study are available within the paper and its supplementary information (SI) files. Supplementary information: supplementary Fig. S1–S8, supplementary table, supplementary methods, and references. See DOI: https://doi.org/10.1039/d5cc06861d.

## References

[cit1] Yi J. J., Wang H., Vilela M., Danuser G., Hahn K. M. (2014). Manipulation of Endogenous Kinase Activity in Living Cells Using Photoswitchable Inhibitory Peptides. ACS Synth. Biol..

[cit2] Renicke C., Schuster D., Usherenko S., Essen L.-O., Taxis C. (2013). A LOV2 Domain-Based Optogenetic Tool to Control Protein Degradation and Cellular Function. Chem. Biol..

[cit3] Mahmoudi P., Veladi H., Pakdel F. G. (2017). Optogenetics, Tools and Applications in Neurobiology, J. Med. Signals Sens..

[cit4] He L., Tan P., Zhu L., Huang K., Nguyen N. T., Wang R., Guo L., Li L., Yang Y., Huang Z., Zhou Y. (2021). Circularly permuted LOV2 as a modular photoswitch for optogenetic engineering. Nat. Chem. Biol..

[cit5] Geng L., Shen J., Wang W. (2021). Circularly permuted AsLOV2 as an optogenetic module for engineering photoswitchable peptides. Chem. Commun..

[cit6] Foight G. W., Wang Z., Wei C. T., Greisen P., Warner K. M., Cunningham-Bryant D., Park K., Brunette T. J., Sheffler W., Baker D., Maly D. (2019). Multi-input chemical control of protein dimerization for programming graded cellular responses. Nat. Biotechnol..

[cit7] Shen J., Geng L., Li X., Emery C., Kroning K., Shingles G., Lee K., Heyden M., Li P., Wang W. (2023). A general method for chemogenetic control of peptide function. Nat. Methods.

[cit8] Miura Y., Senoo A., Doura T., Kiyonaka S. (2022). Chemogenetics of cell surface receptors: beyond genetic and pharmacological approaches. RSC Chem. Biol..

[cit9] Banaszynski L. A., Chen L.-C., Maynard-Smith L. A., Ooi A. G. L., Wandless T. J. (2006). A Rapid, Reversible, and Tunable Method to Regulate Protein Function in Living Cells Using Synthetic Small Molecules. Cell.

[cit10] Hill Z. B., Martinko A. J., Nguyen D. P., Wells J. A. (2018). Human antibody-based chemically induced dimerizers for cell therapeutic applications. Nat. Chem. Biol..

[cit11] Guo Z., Smutok O., Johnston W. A., Walden P., Ungerer J. P. J., Peat T. S., Newman J., Parker J., Nebl T., Hepburn C., Melman A., Suderman R., Katz E., Alexandrov K. (2021). Design of a methotrexate-controlled chemical dimerization system and its use in bio-electronic devices. Nat. Commun..

[cit12] Kang S., Davidsen K., Gomez-Castillo L., Jiang H., Fu X., Li Z., Liang Y., Jahn M., Moussa M., DiMaio F., Gu L. (2019). COMBINES-CID: An Efficient Method for De Novo Engineering of Highly Specific Chemically Induced Protein Dimerization Systems. J. Am. Chem. Soc..

[cit13] Guntas G., Hallett R. A., Zimmerman S. P., Williams T., Yumerefendi H., Bear J. E., Kuhlman B. (2015). Engineering an improved light-induced dimer (iLID) for controlling the localization and activity of signaling proteins. Proc. Natl. Acad. Sci. U. S. A..

[cit14] Lee S.-Y., Cheah J. S., Zhao B., Xu C., Roh H., Kim C. K., Cho K. F., Udeshi N. D., Carr S. A., Ting A. Y. (2023). Engineered allostery in light-regulated LOV-Turbo enables precise spatiotemporal control of proximity labeling in living cells. Nat. Methods.

[cit15] Wang W., Wildes C. P., Pattarabanjird T., Sanchez M. I., Glober G. F., Matthews G. A., Tye K. M., Ting A. Y. (2017). A light- and calcium-gated transcription factor for imaging and manipulating activated neurons. Nat. Biotechnol..

[cit16] Guan X. M., Kobilka T. S., Kobilka B. K. (1992). Enhancement of membrane insertion and function in a type IIIb membrane protein following introduction of a cleavable signal peptide. J. Biol. Chem..

[cit17] Fan F., Binkowski B. F., Butler B. L., Stecha P. F., Lewis M. K., Wood K. V. (2008). Novel Genetically Encoded Biosensors Using Firefly Luciferase. ACS Chem. Biol..

[cit18] Wang F. I., Ding G., Ng G. S., Dixon S. J., Chidiac P. (2022). Luciferase-based GloSensor™ cAMP assay: Temperature optimization and application to cell-based kinetic studies. Methods.

