# Supplementary material for: A novel directed evolution platform for engineering chemically gated protein switches
Source: Chem Commun (Camb). 2026 Feb 24;62(21):5975–9. doi: 10.1039/d5cc06861d (PMC12954488; doi:10.1039/d5cc06861d)
Supplement: CC-062-D5CC06861D-s001 [file CC-062-D5CC06861D-s001.pdf]

Supplementary Information (SI) for ChemComm.
This journal is © The Royal Society of Chemistry 2026

Supporting Information

A Novel Directed Evolution Platform for Engineering Chemically Gated Protein Switches

Luis A. Vázquez-Rivera [a,b#], *Jiaqi Shen*[a,c#], *Gwendolyn Shingles* [a,c], *Guanwei Zhou* [a,b], *Wenjing Wang* [a,b,c,d,e]*

[#]These authors contributed equally
[a] Life Sciences Institute, University of Michigan, Ann Arbor, MI
[b] Program in Chemical Biology, University of Michigan, Ann Arbor, MI
[c] Department of Chemistry, University of Michigan, Ann Arbor, MI
[d] Department of Biologic and Materials Sciences & Prosthodontics, University of Michigan, Ann Arbor, MI
[e] Department of Molecular and Integrative Physiology, University of Michigan, Ann Arbor, MI

*Corresponding author email: wenjwang@umich.edu

Table of Contents

1. **Supplementary figures**
   Figure S1. Experimental design and flow cytometry data for the sequential labeling competition control.
   Figure S2. Assessment of Strep-PE fluorescence stability upon exposure to hydrogen peroxide (H_2O_2).
   Figure S3. Proof-of-principle study of the DuoSelect platform using known positive and negative cells.
   Figure S4. Examples of the consecutive triple gating strategy.
   Figure S5. SingleSelect platform.
   Figure S6. Sequencing results and FACS characterization of the selected CapC variants using DuoSelect.
   Figure S7. Characterization of evolved CapC variants in HEK293T.
   Figure S8. CapN variant characterization using the mammalian two hybrid system in HEK293T.

2. **Supplementary Table.**
   2.1 Plasmids used in this study
   2.2 Amino acid sequences of plasmids

3. **Supplementary Methods**
   3.1 Yeast experiments
      3.1.1 Yeast library generation
      3.1.2 Yeast culture and protein induction
      3.1.3 Single color labeling
      3.1.4 Flow Cytometry and FACS Selection of CapC and CapN
      3.1.5 Double color labeling (add Shield-1 concentration)
      3.1.6 Prove of principle selection (ratios)

   3.2 HEK293T cells application of CAPs
      3.2.1 HEK cell culture and lentiviral infection CapN
      3.2.2 CapC-based mammalian two hybrid assay with luciferase as a readout in HEK 293T Cells
      3.2.3 Shield-1-controlled opioid signaling in HEK 293T cell
      3.2.4 CapN-based mammalian two hybrid assay with mCherry as a readout
      3.2.5 Shield-1- controlled PAC1R signaling in HEK 293T Cells
      3.2.6 Culture *and Transfection of Human Embryonic Kidney (HEK) 293T Cells for Two-Photon Fluorescence Lifetime Imaging (2pFLIM) experiment*
      3.2.7 *2pFLIM and Image Analysis*
   3.3 AAV preparation and administration
      3.3.1 Concentrated AAV prep
      3.3.2 Stereotactic injection of AAV into the mouse brain
      3.3.3 Shield-1 administration and histology

4. **References**

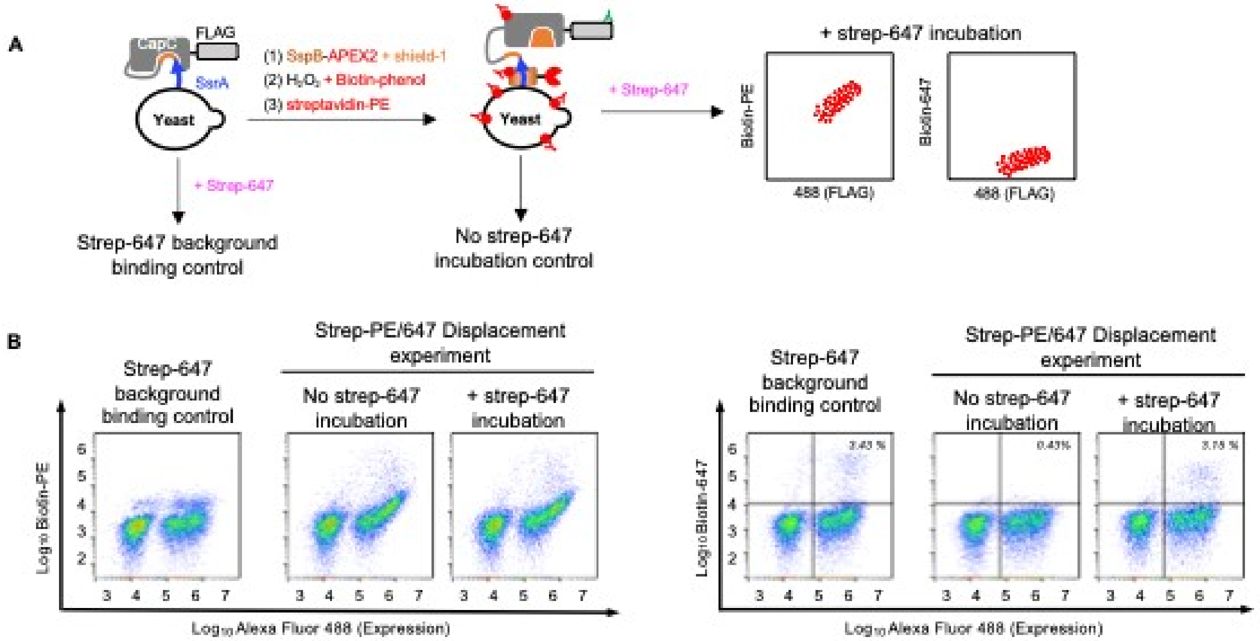

Figure S1. Experimental design and flow cytometry data for the sequential labeling competition control. (A) Schematic of the Strep-PE/647 Displacement Sample workflow. Yeast cells were incubated with SsspB-APEX2 and Shield-1, then biotinylated using H_2O_2 and biotin-phenol. The cells were labeled with saturating Strep-PE followed directly by incubation with Strep-647 without an intermediate activation step. (B) Flow cytometry analysis of the Strep-PE/647 Displacement Sample compared to controls. The Strep-647 background binding control consists of CapC1.1 cells labeled with Strep-647 directly without APEX2 and Shield-1 labeling. (Left) Plot of Strep-PE signal intensity versus surface expression level. (Right) Plot of Strep-647 signal intensity versus surface expression level. Quadrant 2 represents cells positive for Strep-647 signal.

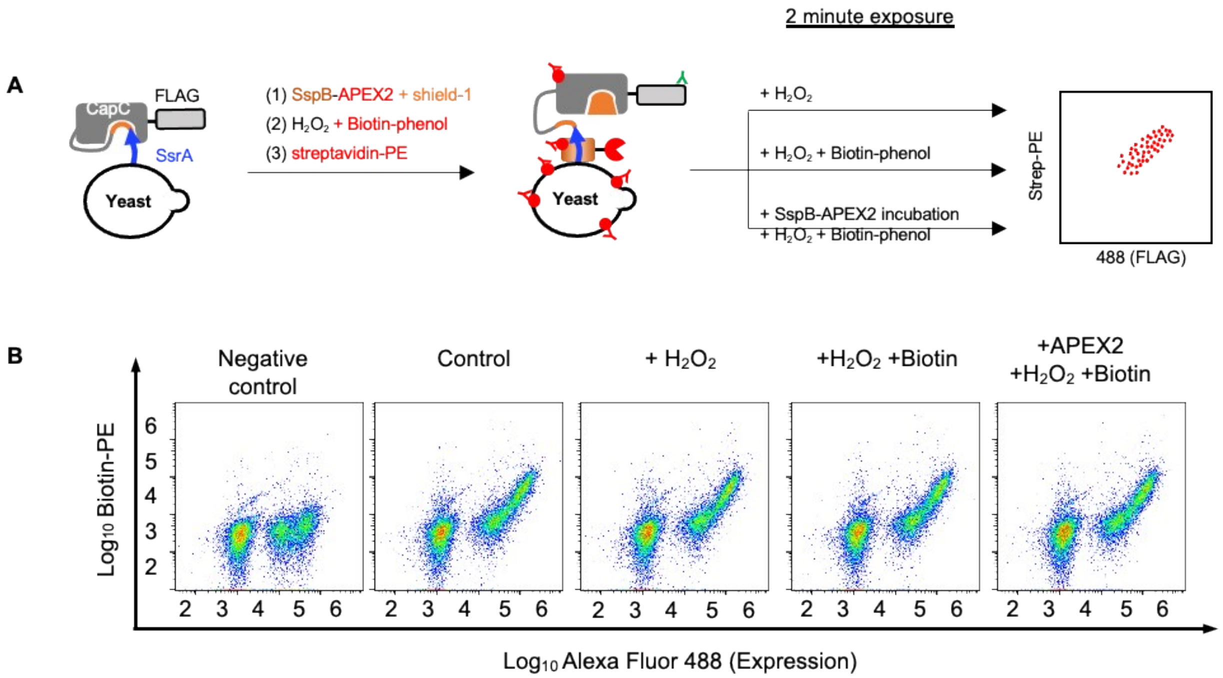

Figure S2. Assessment of Strep-PE fluorescence stability upon exposure to hydrogen peroxide (H_2O_2). (A) Schematic of the control experiment designed to evaluate potential fluorophore quenching with H_2O_2. Cells pre-labeled with Strep-PE were subjected to a 2-minute exposure to reaction components to test signal stability. (B) Flow cytometry analysis comparing Strep-PE fluorescence intensity across five conditions: No drug condition (negative control), a PBSB wash (control), incubation with H_2O_2 alone, incubation with H_2O_2 and biotin, and incubation with APEX followed by the addition of H_2O_2 and biotin.

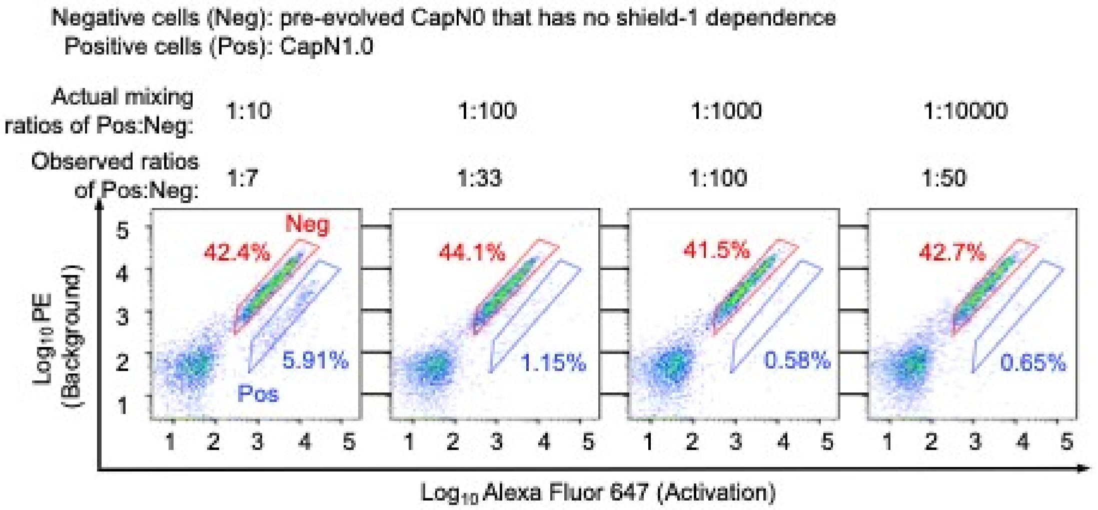

Figure S3. Proof-of-principle study of the DuoSelect platform using known positive and negative cells. The positive and negative cells were mixed with different ratios, followed by DuoSelect labeling and FACS analysis. Negative cells, displaying unevolved CapN0 with no shield-1 dependence. Positive cells, displaying CapN1.0 with shield-1 dependence. The gates on the left and on the right, each represents the negative and the positive cells. The percentages within the FACS plot represents the percentage of cells within the gate compared to the total cells. The observed ratios of Pos:Neg were calculated by calculating the ratios of the percentage of the Pos versus the Neg.

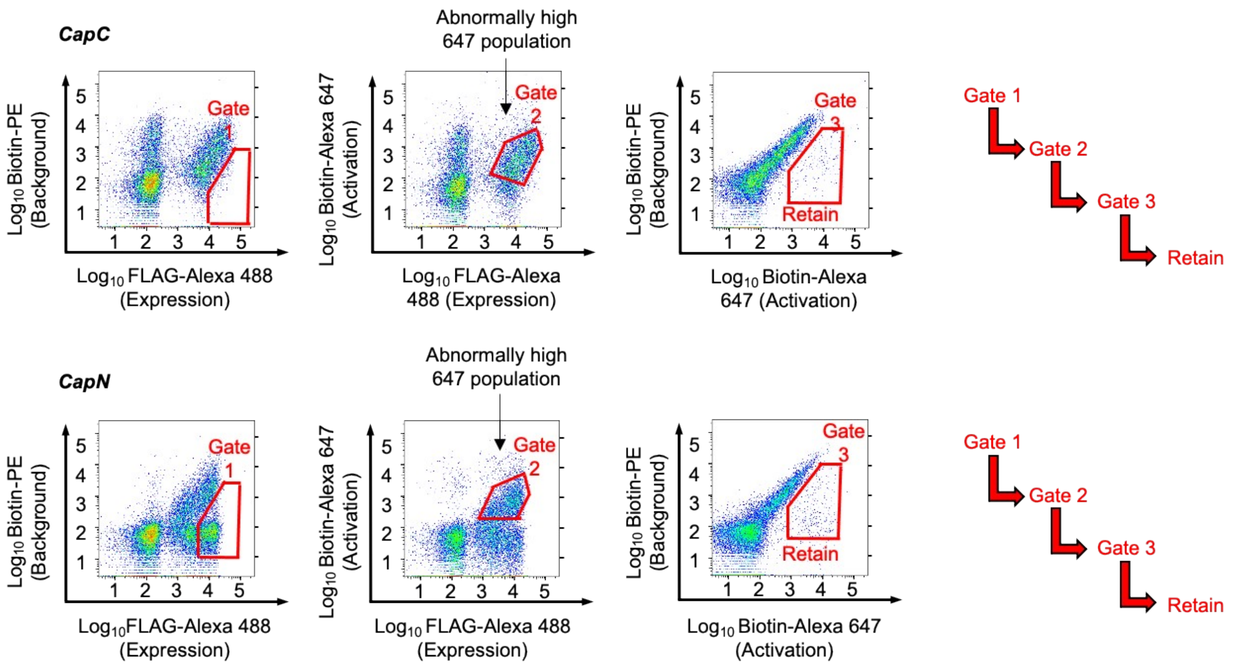

Figure S4. Examples of the consecutive triple gating strategy. Gate 1 was drawn first to select cells with high FLAG but low background signal; gate 2 was drawn within gate 1 to select cells that have high activation signal but represent the main population of cells to avoid the cells with abnormally high 647 signals that causes false positives; gate 3 was drawn lastly within gate 2 to select.

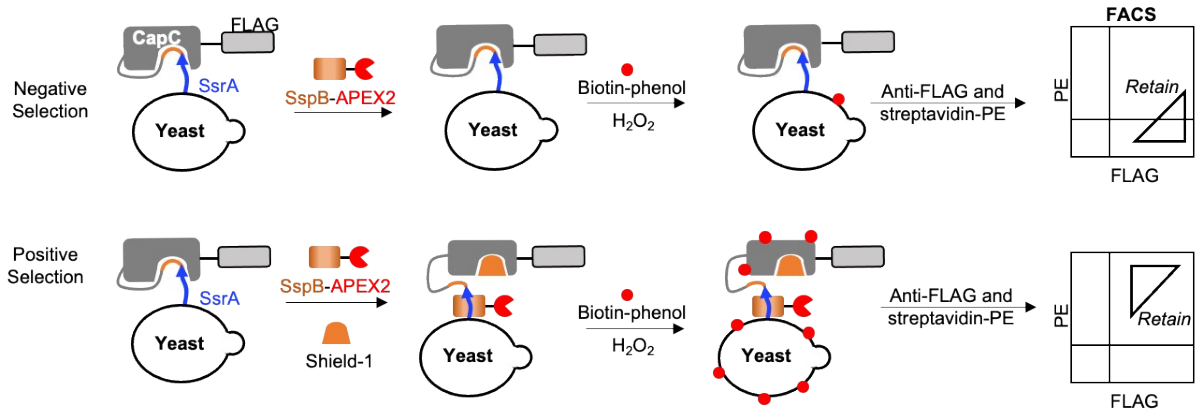

Figure S5. SingleSelect platform. Yeast cells were incubated with SspB-APEX with or without Shield-1, followed by APEX2-dependent proximity labeling with biotin-phenol and H_2O_2. The covalently labeled biotin molecules were further labeled with streptavidin-PE. FLAG tag indicating protein expression.

A

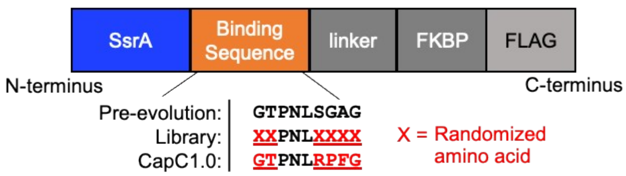

Clone	Frequency/ 24	Sequence
CapC1.1	7	GTPNLFGYV
CapC1.2	4	GSPNLSHRR
CapC1.3	4	GSPNLRASY
CapC1.4	3	GSPNLVPHI
CapC1.5	2	GSPNLRIPD
CapC1.6	1	GFPNLGSWN
CapC1.7	1	GSPNLGVRA
CapC1.8	1	TFPNLNQTD
CapC1.9	1	GFPNLRNVE

B

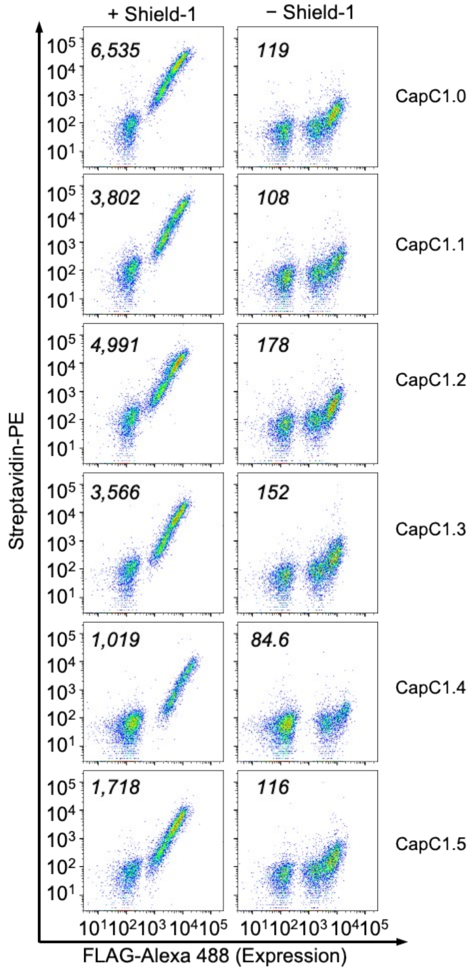

Figure S6. Sequencing results and FACS characterization of the selected CapC variants using DuoSelect. (A) Schematics of the CapC constructs and sequencing results of the enriched CapC variants from Duoselect. Frequency indicates the number of clones among the 24 sequenced clones. (B) FACS analysis characterization of the CapC variants with frequency > 1 in comparison to the previously evolved CapC1.0. The numbers within the FACS plot indicates the medium PE value of the population with positive FLAG signal.

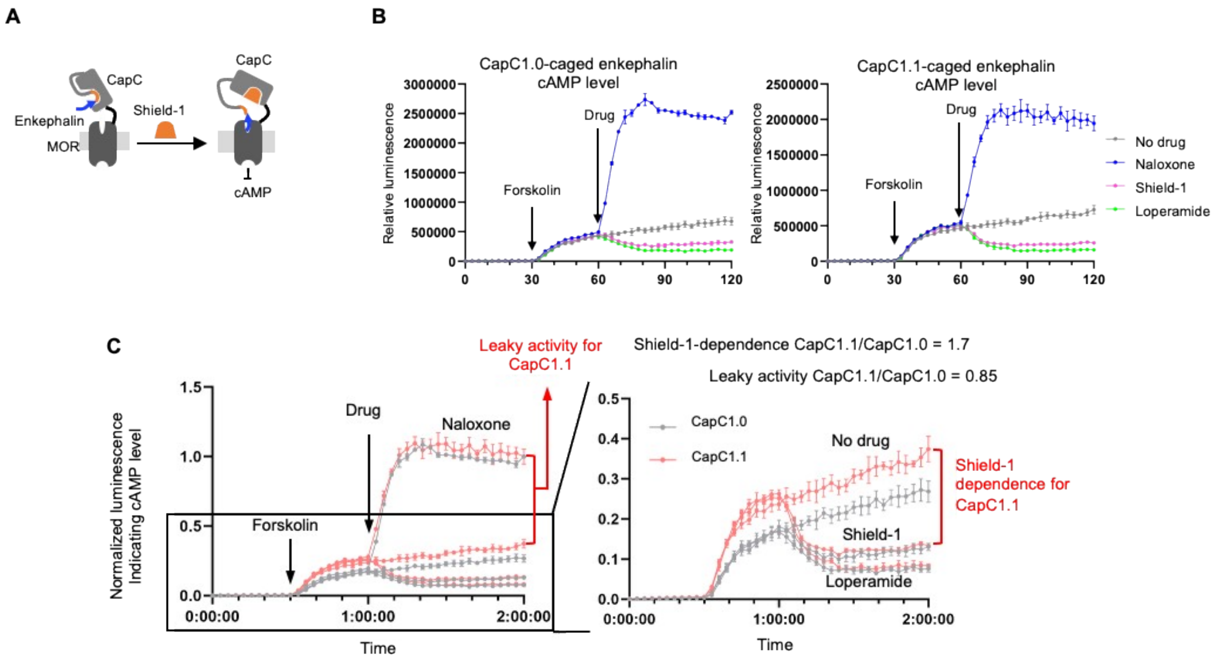

Figure S7. Characterization of evolved CapC variants in HEK293T. (A) Scheme of CapC-controlled enkephalin. Addition of shield-1 uncages enkephalin and activates the MOR, leading to inhibition of cAMP production. A cAMP biosensor, GloSensor was cotransfected to detect cAMP level. (B) Effect of different drugs in the assay shown in A. (B) Cells were first stimulated with forskolin (1 μM) at 30 min, then stimulated with different drugs (10 μM) at 60 min. Left: CapC1.0-caged enkephalin. Right: CapC1.1-caged enkephalin. n = 3 wells from one replicate for all conditions. Errors, s.e.m. (C) Direct comparison of CapC 1.0 and CapC 1.1 by normalizing cAMP level of each construct to the signal of naloxone treated condition at the last time point. Cells were treated with forskolin (1 μM) at 30 min, then stimulated with different drugs (10 μM) at 60 min. cAMP level was read out using GloSensor. Shield-1 dependence was defined as the signal difference between the shield-1 and no drug condition; leaky activity was defined as signal difference between the naloxone and no drug conditions. n = 3 wells from one replicate for all conditions. Errors, s.e.m.

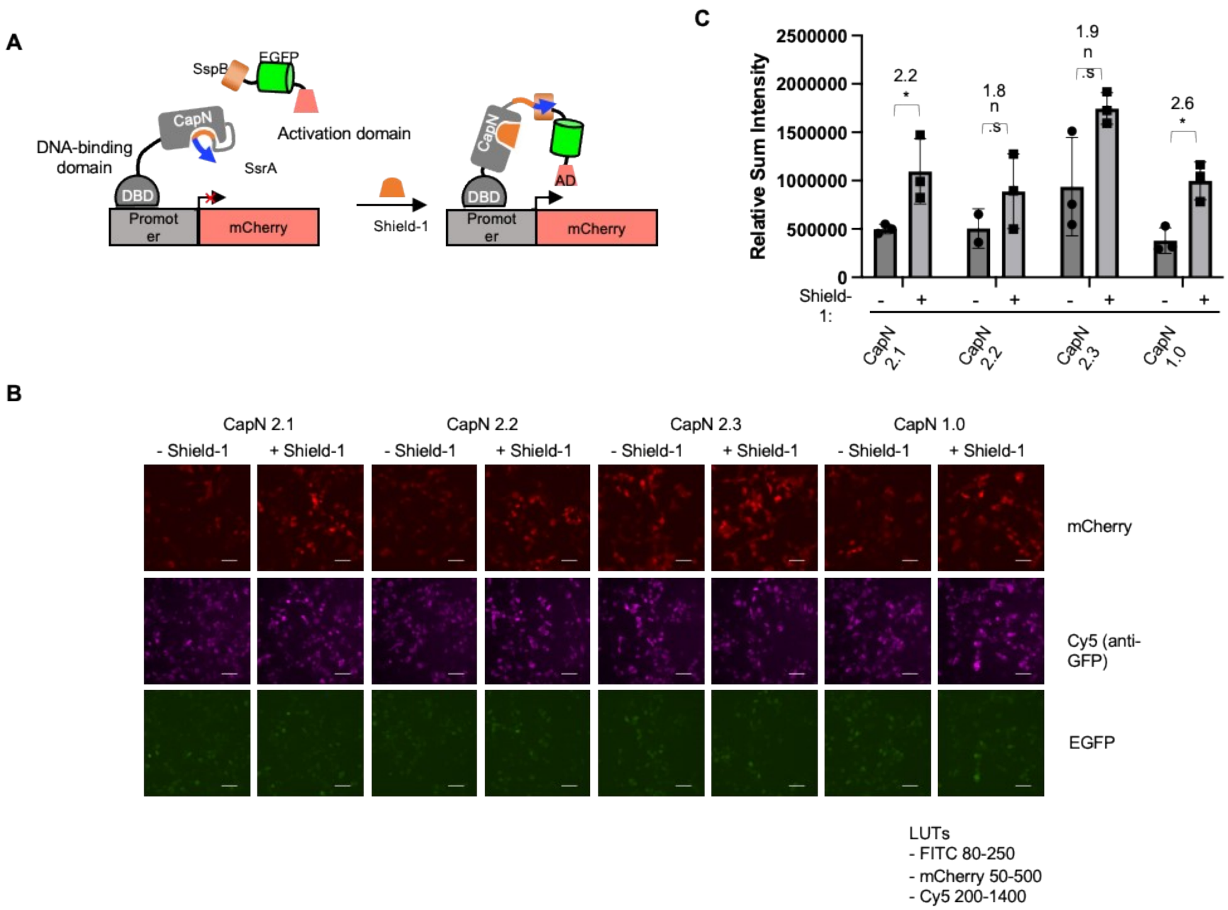

Figure S8. CapN variant characterization using the mammalian two hybrid system in HEK293T. (A) Schematics of the CapN-based mammalian two hybrid system. Similar to the CapC-based system in Figure 2, except that CapN is used for caging SsrA and mCherry is used as the reporter. (B) Representative confocal images of the assay in a. Scale bars, 100 μm. mCherry, reporter expression. Cy5, anti-GFP immunofluorescence. (C) Fluorescence imaging analysis of the CapC-based transcriptional system depicted in a and b under +/- shield-1 conditions. shield-1, 10 μM. The values on the plot are the fluorescence sum intensity signal ratio of the "+ shield-1" versus the "no shield-1" condition. *P* values are determined by unpaired two-tailed *t*-tests. **P* < 0.05; *ns*, not significant. Errors, s.e.m.

2. Supplementary Table.
2.1 Plasmids used in this study

Plasmid Number	Plasmid Name	Vector	5' noncoding region	3' noncoding region	Expression in	Antibiotic resistance	Used in
P1	Aga2p-linker-SsrA-CapC1.1-FLAG	pCTCON2	Gal1/10	-	Yeast	Ampicillin	Fig. S4b
P2	FLAG-GAL4DBD-NLS-SsrA-CapC1.1	plx208	CMV	WPRE-PolyA	Mammalian	Ampicillin	Fig. 2b
P3	SspB-Linker-EGFP-NLS-VP16	plx208	CMV	WPRE-PolyA	Mammalian	Ampicillin	Fig. 2b
P4	Signal Sequence-PACAP(1-23)-Truncated CapC1.1-HA Tag-HA Tag-Human CD4-CIBN-P2A-mCherry	pAAV	CMV	PolyA	Mammalian	Ampicillin	Fig. 2b
P5	Signal Sequence-Truncated CapC1.1-HA Tag-HA Tag-Human CD4-CIBN-P2A-mCherry	pAAV	CMV	PolyA	Mammalian	Ampicillin	Fig. 2b
P6	Aga2p-linker-FLAG-CapN2.1-SsrA	pCTCON2	Gal1/10	-	Yeast	Ampicillin	Fig. 3d
P7	Aga2p-linker- FLAG-CapN2.2-SsrA	pCTCON2	Gal1/10	-	Yeast	Ampicillin	Fig. 3d
P8	Aga2p-linker- FLAG-CapN2.3-SsrA	pCTCON2	Gal1/10	-	Yeast	Ampicillin	Fig. 3d
P9	FLAG-Gal4 DBD-NLS-CapN2.1-SsrA	plx208	CMV	WPRE-PolyA	Mammalian	Ampicillin	Fig. S6d
P10	FLAG-Gal4 DBD-NLS-CapN2.2-SsrA	plx208	CMV	WPRE-PolyA	Mammalian	Ampicillin	Fig. S6d
P11	FLAG-Gal4 DBD-NLS-CapN2.3-SsrA	plx208	CMV	WPRE-PolyA	Mammalian	Ampicillin	Fig. S6d

2.2 Amino acid sequences of plasmids

P1: Aga2p-linker-<u>SsrA</u>-**CapC1.1**-*FLAG*
MQLLRCFSIFSVIASVLAQELTTICEQIPSPTLESTPYSLSTTTILANGKAMQGVFEYYKSVTFVSNCGSHPS
TTSKGSPINTQYVFKDNSSTIEGRGGSGSGNDSTDGSALQQGLQASGGGGSGAS<u>AANDENYF</u>**GTPNLF**
GYVSGGSGTGSGSGGSGVQVETISPGDGRTFPKRGQTCVVHYTGMLEDGKKVDSSRDRNKPFKFML
GKQEVIRGWEEGVAQMSVGQRAKLTISPDYAYGATGHPGIIPPHATLVFDVELLKLEGS*DYKDDDDK*

P2: *FLAG*-GAL4DBD-**NLS**-<u>SsrA</u>-**CapC1.1**
M*DYKDDDDK*KLLSSIEQACDICRLKKLKCSKEKPKCAKCLKNNWECRYSPKTKRSPLTRAHLTEVESRLE
RLEQLFLLIFPREDLDMILKMDSLQDIKALLTGLFVQDNVNKDAVTDRLASVETDMPLTLRQHRISATSSSE
ESSNKGQRQLTVS**PKKKRKV**AS<u>AANDENY</u>**GTPNLFGYVSGGSGTGSGSGGSGVQVETISPGDGRTFP**

KRGQTCVVHYTGMLEDGKKVDSSRDRNKPFKFMLGKQEVIRGWEEGVAQMSVGQRAKLTISPDYAYGATGHPGIIPPHATLVFDVELLKLE

P3: SspB-Linker-EGFP-NLS-**VP16**

MSQLTPRRPYLLRAFYEWLLDNQLTPHLVVDVTLPGVQVPMEYARDGQIVLNIAPRAVGNLELANDEVRFNARFGGIPRQVSVPLAAVLAIYARENGAGTMFEPEAAYDGSGGGMVSKGEELFTGVVPILVELDGDVNGHKFSVSGEGEGDATYGKLTLKFICTTGKLPVPWPTLVTTLTYGVQCFSRYPDHMKQHDFFKSAMPEGYVQERTIFFKDDGNYKTRAEVKFEGDTLVNRIELKGIDFKEDGNILGHKLEYNYNSHNVYIMADKQKNGIKVNFKIRHNIEDGSVQLADHYQQNTPIGDGPVLLPDNHYLSTQSALSKDPNEKRDHMVLLEFVTAAGITLGMDELYKPKKKRKV**APPTDVSLGDELHLDGEDVAMAHADALDDFDLDMLGDGDSPGPGFTPHDSAPYGALDMADFEFEQMFTDALGIDEYGG**

P4: *Signal Sequence*-PACAP(1-23)-**Truncated CapC1.1**-*HA Tag*-HA Tag-Human CD4-**CIBN**-*P2A*-mCherry

M*KTIIALSYIFCLVFA*HSDGIFTDSYSRYRKQMAVKKYL**PNLFGYVSGGSGTGSGSGGSGVQVETISPGDGRTFPKRGQTCVVHYTGMLEDGKKVDSSRDRNKPFKFMLGKQEVIRGWEEGVAQMSVGQRAKLTISPDYAYGATGHPGIIPPHATLVFDVELLKLE**RS*YPYDVPDYA*YPYDVPDYALDFQKASSIVYKKEGEQVEFSFPLAFTVEKLTGSGELWWQAERASSSKSWITFDLKNKEVSVKRVTQDPKLQMGKKLPLHLTLPQALPQYAGSGNLTLALEAKTGKLHQEVNLVVMRATQLQKNLTCEVWGPTSPKLMLSLKLENKEAKVSKREKAVWVLNPEAGMWQCLLSDSGQVLLESNIKVLPTWSTPVQPMALIVLGGVAGLLLFIGLGIFFCVRCRHRRRKGSGSTSGSGSGGSRGSGGSSGG**MNGAIGGDLLLNFPDMSVLERQRAHLKYLNPTFDSPLAGFFADSSMITGGEMDSYLSTAGLNLPMMYGETTVEGDSRLSISPETTLGTGNFKAAKFDTETKDCNEAAKKMTMNRDDLVEEGEEEKSKITEQNNGSTKSIKKMKHKAKKEENNFSNDSSKVTKELEKTDYIH***GSGATNFSLLKQAGDVEENPGP*TGARDPPVATMVSKGEEDNMAIIKEFMRFKVHMEGSVNGHEFEIEGEGEGRPYEGTQTAKLKVTKGGPLPFAWDILSPQFMYGSKAYVKHPADIPDYLKLSFPEGFKWERVMNFEDGGVVTVTQDSSLQDGEFIYKVKLRGTNFPSDGPVMQKKTMGWEASSERMYPEDGALKGEIKQRLKLKDGGHYDAEVKTTYKAKKPVQLPGAYNVNIKLDITSHNEDYTIVEQYERAEGRHSTGGMDELYK

P5: *Signal Sequence*-**Truncated CapC1.1**-*HA Tag*-HA Tag-Human CD4-**CIBN**-*P2A*-mCherry

M*KTIIALSYIFCLVFA***PNLFGYVSGGSGTGSGSGGSGVQVETISPGDGRTFPKRGQTCVVHYTGMLEDGKKVDSSRDRNKPFKFMLGKQEVIRGWEEGVAQMSVGQRAKLTISPDYAYGATGHPGIIPPHATLVFDVELLKLE**RS*YPYDVPDYA*YPYDVPDYALDFQKASSIVYKKEGEQVEFSFPLAFTVEKLTGSGELWWQAERASSSKSWITFDLKNKEVSVKRVTQDPKLQMGKKLPLHLTLPQALPQYAGSGNLTLALEAKTGKLHQEVNLVVMRATQLQKNLTCEVWGPTSPKLMLSLKLENKEAKVSKREKAVWVLNPEAGMWQCLLSDSGQVLLESNIKVLPTWSTPVQPMALIVLGGVAGLLLFIGLGIFFCVRCRHRRRKGSGSTSGSGSGGSRGSGGSSGG**MNGAIGGDLLLNFPDMSVLERQRAHLKYLNPTFDSPLAGFFADSSMITGGEMDSYLSTAGLNLPMMYGETTVEGDSRLSISPETTLGTGNFKAAKFDTETKDCNEAAKKMTMNRDDLVEEGEEEKSKITEQNNGSTKSIKKMKHKAKKEENNFSNDSSKVTKELEKTDYIH***GSGATNFSLLKQAGDVEENPGP*TGARDPPVATMVSKGEEDNMAIIKEFMRFKVHMEGSVNGHEFEIEGEGEGRPYEGTQTAKLKVTKGGPLPFAWDILSPQFMYGSKAYVKHPADIPDYLKLSFPEGFKWERVMNFEDGGVVTVTQDSSLQDGEFIYKVKLRGTNFPSDGPVMQKKTMGWEASSERMYPEDGALKGEIKQRLKLKDGGHYDAEVKTTYKAKKPVQLPGAYNVNIKLDITSHNEDYTIVEQYERAEGRHSTGGMDELYK

P6: Aga2p-linker-*FLAG*-**CapN2.1**-SsrA

MQLLRCFSIFSVIASVLAQELTTICEQIPSPTLESTPYSLSTTTILANGKAMQGVFEYYKSVTFVSNCGSHPSTTSKGSPINTQYVFKDNSSTIEGRGGSGSGNDSTDGSALQQGLQASGGGGSGAS*DYKDDDDK***TSGVQVETISPGDGRTFPKRGQTCVVHYTGMLEDGKKVDSSRDRNKPFKFMLGKQEVIRGWEEGVAQMSVGQRAKLTISPDYAYGATGHPGIIPPHATLVFDVELLKLETRGVEEVERYMPNL**AANDENYF

P7: Aga2p-linker-*FLAG*-**CapN2.2**-SsrA

MQLLRCFSIFSVIASVLAQELTTICEQIPSPTLESTPYSLSTTTILANGKAMQGVFEYYKSVTFVSNCGSHPSTTSKGSPINTQYVFKDNSSTIEGRGGSGSGNDSTDGSALQQGLQASGGGGSGAS*DYKDDDDK***TSGVQVETISPGDGRTFPKRGQTCVVHYTGMLEDGKKVDSSRDRNKPFKFMLGKQEVIRGWEEGVAQMSVGQRAKLTISPDYAYGATGHPGIIPPHATLVFDVELLKLETRGVEEVERYSPNL**AANDENYF

P8: Aga2p-linker-*FLAG*-**CapN2.3**-SsrA
MQLLRCFSIFSVIASVLAQELTTICEQIPSPTLESTPYSLSTTTILANGKAMQGVFEYYKSVTFVSNCGSHPSTTSKGSPINTQYVFKDNSSTIEGRGGSGSGNDSTDGSALQQGLQASGGGGSGAS*DYKDDDDK***TSGVQVETISPGDGRTFPKRGQTCVVHYTGMLEDGKKVDSSRDRNKPFKFMLGKQEVIRGWEEGVAQMSVGQRAKLTISPDYAYGATGHPGIIPPHATLVFDVELLKLETRGVEEVYRGSPNL**AANDENYF

P9: *FLAG-Gal4 DBD*-NLS-**CapN2.1**-SsrA
M*DYKDDDDKKLLSSIEQACDICRLKKLKCSKEKPKCAKCLKNNWECRYSPKTKRSPLTRAHLTEVESRLERLEQLFLLIFPREDLDMILKMDSLQDIKALLTGLFVQDNVNKDAVTDRLASVETDMPLTLRQHRISATSSSEESSNKGQRQLTVS*PKKKRKVAS**GVQVETISPGDGRTFPKRGQTCVVHYTGMLEDGKKVDSSRDRNKPFKFMLGKQEVIRGWEEGVAQMSVGQRAKLTISPDYAYGATGHPGIIPPHATLVFDVELLKLETRGVEEVERYMPNL**AANDENYF

P10: *FLAG-Gal4 DBD*-NLS-**CapN2.2**-SsrA
M*DYKDDDDKKLLSSIEQACDICRLKKLKCSKEKPKCAKCLKNNWECRYSPKTKRSPLTRAHLTEVESRLERLEQLFLLIFPREDLDMILKMDSLQDIKALLTGLFVQDNVNKDAVTDRLASVETDMPLTLRQHRISATSSSEESSNKGQRQLTVS*PKKKRKVAS**GVQVETISPGDGRTFPKRGQTCVVHYTGMLEDGKKVDSSRDRNKPFKFMLGKQEVIRGWEEGVAQMSVGQRAKLTISPDYAYGATGHPGIIPPHATLVFDVELLKLETRGVEEVERYSPNL**AANDENYF

P11: *FLAG-Gal4 DBD*-NLS-CapN2.3-SsrA
M*DYKDDDDKKLLSSIEQACDICRLKKLKCSKEKPKCAKCLKNNWECRYSPKTKRSPLTRAHLTEVESRLERLEQLFLLIFPREDLDMILKMDSLQDIKALLTGLFVQDNVNKDAVTDRLASVETDMPLTLRQHRISATSSSEESSNKGQRQLTVS*PKKKRKVAS**GVQVETISPGDGRTFPKRGQTCVVHYTGMLEDGKKVDSSRDRNKPFKFMLGKQEVIRGWEEGVAQMSVGQRAKLTISPDYAYGATGHPGIIPPHATLVFDVELLKLETRGVEEVYRGSPNL**AANDENYF

3. Supplementary Methods

3.1 Yeast experiments

3.1.1 Yeast library generation

CapN Yeast library generation was following the previous protocol[1]. Briefly, the CapN gene fragment were randomized in the binding sequences area through targeted mutagenesis using standard PCR techniques and degenerated primers from IDT. The linearized vector (2 µg) mixed with 8 µg of the randomized PCR fragments were concentrated using pellet paint (Millipore Sigma, #70748) and then electroporated into home made yeast competent cells. The library size for CapN was estimated to be approximately 1 x 10^8.

3.1.2 Yeast culture and protein induction

Yeast cell culture protocols were adapted from previous work[1]. Yeast was grown in SDCAA media (2% dextrose (Thermo Fisher Scientific, #D16-1), 0.67 yeast nitrogen base without amino acids (Bio Basic, #S507), 0.5% Casamino acids (Thermo Fisher, #223120), 0.54% Disodium phosphate, 0.856% Monosodium phosphate) at 30 °C with shaking at 220 r.p.m. To induce expression of the construct, 500 µl yeast in SDCAA medium was added to 5 ml SGCAA (same recipe as SDCAA but 2% dextrose was replaced with 2% galactose (Thermo Fisher Scientific, #BP656)) and let grow overnight.

3.1.3 Single color labeling

Yeast single color labeling protocols were similar to previous work[1]. Briefly, for labeling, 250 µl (or 1 ml for the first round of library selection) of yeast induced overnight was washed with 1 ml of PBSB (sterile phosphate-buffered saline supplemented with 0.1% bovine serum albumin (BSA) (DOT, #DSA30075)) twice before labeling. Yeast expressing either CapC or CapN caged SsrA were incubated with SspB-APEX2 (200 µL, 0.24 mg/mL in PBSB) with or without shield-1 (10 µM, AOBIOUS, Reference #AOB1848) at RT for 30 minutes under rotation. Yeast was washed twice with 1 mL PBSB, followed by APEX reaction in 950 uL of 1% BSA (in PBSB) with biotin-phenol (1 µM) and hydrogen peroxide (~10 µM). Then, the yeast were immunostained with mouse anti-FLAG antibodies (Sigma, Reference #F3165, followed by anti-mouse-

AlexaFluoro647, Life Technologies, References #A-21235) and streptavidin fused with PE(Jackson ImmunoResearch, Reference #016-110-084).

3.1.4 Flow Cytometry and FACS Selection of CapC and CapN

The FACS Aria III cell sorter flow cytometer (BD Biosciences) was used to sort all labeled cells. The equipment was outfitted with a 633 nm laser and 660/20 emission filter along with a 561 nm laser and a 582/15 emission filter. Subsequently, they were subject to further analysis. For conventional selection for CapC; round 1 (negative selection): 0.66% (1.3 × 10^7 cells); round 2 (positive selection): 0.01% (6.1 × 10^6 cells); round 3: 0.04% (4.4 × 10^6 cells). In CapC DuoSelect evolution: round 1: 0.02% (4.0 × 10^7 cells); round 2: 0.01% (4.0 × 10^5 cells); round 3: 0.001% (9.0 × 10^6 cells). Numbers of cells for CapN DuoSelect was as followed: round 1: 0.09% from Library 1 (8.6 × 10^6 cells); round 2: 0.07% (9.1 × 106 cells); round 3: 0.72% from Library 3 (1.0 × 10^7 cells). The sorted yeast cells were then gathered into 5 ml SDCAA medium complemented with 1% penicillin–streptomycin (Thermo Fisher, #15140122), and 30 μg ml−1 kanamycin (Bio Basic, #KB0286). The cells were then incubated at 30 °C and subjected to shaking at 220 r.p.m. for a duration of 2–3 days right after sorting.

3.1.5 Double color labeling (add Shield-1 concentration)

Yeast cells displaying CapC or CapN caged SsrA were incubated with 200 uL of 0.24 mg/mL of SspB-APEX2 (ascorbate peroxidase) for 30 minutes dissolved in PBSB (0.1 % BSA in PBS). SspB will only bind to the accessible SsrA (either leaky or uncaged) and bring the APEX to the proximity of the yeast cells. Then SspB- APEX labeling was performed without addition of shield-1 in the first step to capture the leaky SsrA binding to SspB- APEX, and was left incubating for 30 minutes. After incubation, yeast was washed twice with 1 mL PBSB and then 950 uL of 1% BSA in PBSB was added to the yeast. Further incubation of the bound APEX with 1 μL of 0.1 μM concentration of biotin-phenol and 1 μl of 9.3 mM concentration of hydrogen peroxide will generate a reactive biotin-phenol radical species that can covalently label nearby proteins with biotin which will be further labeled with Streptavidin (Strep)-PE.

Then, shield-1 (final concentration: 10 μM) was used to uncage SsrA and perform the SspB-APEX labeling again but label the biotin with Strep- 647 in this step. Therefore, strep-PE signal indicates the leakiness of the caged SsrA, while strep-647 indicates the uncaging efficiency of CapC. The goal was to select CapC variants with high strep-647 (uncaging) and low strep-PE (leaky activity).

3.1.6 Prove of principle selection (ratios)

To evaluate the efficacy of the DuoSelect platform, the first step is to obtain two distinct yeast samples: the evolved CapN 1.0 and the original CapN. The OD600nm of the samples were takn to ensure that the cultures are growing similary and to facilitate the mixing of the different yeasts. Once the cultures are grown to a sufficient level, samples can then be prepared by combining the two yeast cultures in varying proportions. Begin with a 1:10 ratio, where for every unit of CapN 1.0, ten units of the original CapN strain are added. Subsequently increase the proportion of the original CapN in the mixture according to the ratios 1:100, 1:1000, and finally, 1:10000. Ensure the proportion of CapN 1.0 remains constant in each mixture. After mixing, APEX double color labeling was done as described above. The samples were later analyzed by flow cytometry on the LSR Fortessa cell analyzer flow cytometer BD Biosciences.

3.2 HEK293T cells application of CAPs

3.2.1 HEK cell culture and lentiviral infection CapN

The protocols for HEK 293T cell (ATCC, #CRL-11268) culturing were adapted from previous protocol[1]. Briefly, HEK 293T cells with fewer than 20 passages were grown in an incubator at 37 °C with 5% CO_2 with complete growth medium (DMEM (Fisher, #11-885-092) and MEM (Fisher, #11-095-098) were mixed in a 1:1 ratio, with 10% fetal bovine serum (Biowest, #S1620), 20 mM of HEPES (Thermo Fisher, #15630080), and 1% of penicillin–streptomycin). HEK293T cells were checked against mycoplasma contamination frequently through DAPI staining and cell health were evaluated based on morphology, cell replication speed, and transfection efficacy of the cells.

For lentiviral production, HEK 293T cells were mixed with premixed DNA-PEI mix (2.5 μg of viral DNA, 0.25 μg of pVSVG, and 2.25 μg of Δ8.9 lentiviral guide plasmid) and plated at 80% confluence in a T25 cell culture flask. The cells were grown for an additional 36-48 hours before the supernatant lentivirus were gathered, flash frozen in liquid nitrogen, and store at −80 °C.

3.2.2 CapC-based mammalian two hybrid assay with luciferase as a readout in HEK 293T Cells

HEK 293T cells were plated in 96-well solid white polystyrene microplates (Fisher, #07-200-589) at 50% confluency and then transduced with 33 μL lentivirus supernatant, each, of UAS-Luciferase, SspB-EGFP-

VP16, and Gal4BD-CapC constructs. Three wells of cells for each condition were infected. The HEK293T cells were incubated for 24 hours at 37°C and then aspirated to replace the media with 100 µL of new complete media and 100 µL of complete media with 10 µM shield-1 for with drug conditions. The cells where then left to stimulate gene transcription for luciferase for an additional 24 hours at 37°C. The media was then aspirated and 50 µL BrightGlo (Promega) was added to the wells and incubated at room temperature for 5 minutes. The luminescent intensity from luciferase was measured with a BioTek Cytation 5 Cell Imaging Multireader (Agilent) using the autogain feature to determine the optimal amount of time readout for luminescence within the plate readers detection limit. Only a single luminescent readout was measured for each well. Further analysis and plotting was done using GraphPad Prism10.

3.2.3 Shield-1-controlled opioid signaling in HEK 293T cell

For each well in a 96-well white bottom plate, HEK 293T cells at 90% confluence were transfected with 75ng of GloSensor and 150ng mOR plasmids. For each condition, three wells were prepared. After 24hr, the old media was replaced by 100 uL 2 mM d-luciferin potassium salt (Gold Bio, #LUCK) in complete growth medium (with 50 mM HEPES). BioTek CYTATION 5 plate reader was used to measure the luminescence. After 30min, the baseline luminescence should reach equilibration and cells were treated with 1 µl 100 µM forskolin (Sigma-Aldrich, #F6886). At 60min, 1 µl 1 mM drugs including naloxone (Sigma-Aldrich, #N7758), shield-1, and DAMGO (Sigma-Aldrich, #E7384), respectively, was added to different wells.

3.2.4CapN-based mammalian two hybrid assay with mCherry as a readout

For the CapN-based mammalian two hybrid (MTH) experiments, HEK 293T cells were infected with the lentiviral mix of UAS-mCherry, GAL4BD-CapN, and SspB-EGFP-VP16 in 6-well plates at 40% confluence. The cells were grown for 24 hours prior to stimulation with or without shield-1 (10 µM). The cells were then replated into the 96-well solid white polystyrene microplates (Fisher, #07-200-589) at a 50% confluency in triplicates per condition and grown for 24 hours. Then, the cells were fixed and immunostained against EGFP before fluorescence imaging. The cells were analyzed following previous protocols[1] using Nikon NIS-Elements analysis module and Prism 10.

4. References:

(1) Shen, J.; Geng, L.; Li, X.; Emery, C.; Kroning, K.; Shingles, G.; Lee, K.; Heyden, M.; Li, P.; Wang, W. A general method for chemogenetic control of peptide function. *Nat Methods* **2023**, *20* (1), 112-122. DOI: 10.1038/s41592-022-01697-8 From NLM Medline.

(2) Chen, Y.; Saulnier, J. L.; Yellen, G.; Sabatini, B. L. A PKA activity sensor for quantitative analysis of endogenous GPCR signaling via 2-photon FRET-FLIM imaging. *Front Pharmacol* **2014**, *5*, 56. DOI: 10.3389/fphar.2014.00056 From NLM PubMed-not-MEDLINE.

(3) Ma, P.; Chen, P.; Tilden, E.; Aggarwal, S.; Oldenborg, A.; Chen, Y. Fluorescence lifetime enables high-resolution analysis of neuromodulator dynamics across time and animals. *bioRxiv* **2023**, 2022.2009.2028.510014. DOI: 10.1101/2022.09.28.510014.

(4) Wang, W.; Wildes, C. P.; Pattarabanjird, T.; Sanchez, M. I.; Glober, G. F.; Matthews, G. A.; Tye, K. M.; Ting, A. Y. A light- and calcium-gated transcription factor for imaging and manipulating activated neurons. *Nat Biotechnol* **2017**, *35* (9), 864-871. DOI: 10.1038/nbt.3909 From NLM Medline.
